# Role of Serum Cholesterol and Statin Use in the Risk of Prostate Cancer Detection and Tumor Aggressiveness

**DOI:** 10.3390/ijms150813615

**Published:** 2014-08-06

**Authors:** Juan Morote, Anna Celma, Jacques Planas, José Placer, Inés de Torres, Mireia Olivan, Juan Carles, Jaume Reventós, Andreas Doll

**Affiliations:** 1 Department of Urology, Vall d’Hebron University Hospital and Autonomous University of Barcelona, Barcelona 08035, Spain; E-Mails: acelma@vhebron.net (A.C.); jplanas@vhebron.net (J.P.); jplacer@vhebron.net (J.P.); itorres@vhebron.net (I.T.); 2 Department of Pathology, Vall d’Hebron University Hospital and Autonomous University of Barcelona, Barcelona 08035, Spain; 3 Department of Medical Oncology, Vall d’Hebron University and Hospital Autonomous University of Barcelona, Barcelona 08035, Spain; E-Mail: jocarles@vhebron.net; 4 Research Unit in Biomedicine and Translational Oncology, Vall d’Hebron Research Institute and Hospital and Autonomous University of Barcelona, Barcelona 08035, Spain; E-Mails: mireia.olivan@vhir.org (M.O.); jaume.reventos@vhir.org (J.R.); andreas.doll@vhir.org (A.D.); 5 Department of Basic Sciences, International University of Catalonia, Barcelona 08017, Spain; 6 IDIBELL-Bellvitge Biomedical Research Institute, Hospitalet de Llobregat, Barcelona 08908, Spain

**Keywords:** statins, cholesterol, prostate cancer risk, prostate cancer aggressiveness

## Abstract

The aim of this study was to analyze the relationship between statin use along with serum cholesterol levels and prostate cancer (PCa) detection and aggressiveness. Statin users of three years or more and serum cholesterol levels (SC) were assessed in 2408 men scheduled for prostate biopsy. SC was classified as normal (NSC: <200 mg/dL) or high (HSC: >200 mg/dL). High-grade PCa (HGPCa) was considered if the Gleason score was greater than 7. Statin users comprised 30.9% of those studied. The PCa detection rate was 31.2% of men on statins and 37% of non-statin users (*p* < 0.006). The PCa detection rate was 26.3% in men with NSC and 40.6% in those with HSC (*p* < 0.001). In the subset of NSC men, the PCa rate was 26.5% for statin users and 26.2% for non-users (*p* = 0.939), while in men with HSC, the PCa rate was 36.4% for statin users and 42.0% for non-statin users (*p* = 0.063). The HGPCa rate was 41.8% for statin users and 32.5% for non-users (*p* = 0.012). NSC men had a 53.8% rate of HGPCa, while the rate was only 27.6% in HSC men (*p* < 0.001). NSC men on statins had an HGPCa rate of 70.2%, while non-statin users had a rate of 41.2% (*p* < 0.001). The HGPCa rate for HSC men on statins was 18.8%, while the rate was 30.0% (*p* = 0.011) for non-users. Logistic regression analysis suggested that serum cholesterol levels could serve as an independent predictor of PCa risk, OR 1.87 (95% CI 1.56–2.24) and HGPCa risk, OR 0.31 (95% CI 0.23–0.44), while statin usage could not. Statin treatment may prevent PCa detection through serum cholesterol-mediated mechanisms. A disturbing increase in the HGPCa rate was observed in statin users who normalized their serum cholesterol.

## 1. Introduction

Prostate cancer (PCa) is the most commonly diagnosed neoplasm among men in Europe and the US [1,2]. In addition, within the same geographic areas, hypercholesterolemia is one of the most frequent conditions observed in men at the age of PCa risk [3]. Statins (HMG-CoA reductase inhibitors), a class of cholesterol-lowering drugs, are the second most prescribed therapeutic medication after painkillers [4]. Therefore, examining the relationship between PCa, statins use and serum cholesterol would seem reasonable, especially taking into consideration recent findings in the literature.

The relationship between serum cholesterol and the incidence of PCa and its mortality have been studied by several groups with inconsistent results. Some studies found a positive association between serum cholesterol and PCa mortality [5,6], while others revealed either an inverse relationship [7,8] or no overall association with the incidence of PCa [9,10,11,12,13]. Five recent reports suggested that men with low serum cholesterol are less likely to have high-grade PCa, although in those studies, serum cholesterol was not associated with the incidence of PCa [11,12,13,14,15]. On the other hand, several epidemiological studies investigated the association between statins use and the overall incidence of cancer. These studies were generally designed to investigate the effects of statins on cardiovascular disease, usually with cancer incidence as a secondary endpoint or adverse effect finding [16]. Several recent studies have examined the direct relationship between statins and PCa incidence, and some of them have shown an inverse relationship between the use of statins and PCa risk [17,18,19,20,21]. In contrast, other studies reported largely inconclusive or negative results when correlating statin use and PCa risk [4,22]. Finally, other studies have related the use of statins to a reduced risk of advanced or aggressive PCa [4,13,22,23,24,25]. The mechanisms by which statins may influence prostate carcinogenesis could be mediated through serum cholesterol reduction and its influence on testosterone synthesis. It is also possible that they have direct anti-tumor action through the inhibition of Ras superfamily isoprenylation and its apoptotic action [26].

The aim of our study was to explore whether the reduction of serum cholesterol through statin use influences the risk of PCa detection and tumor aggressiveness.

## 2. Results and Discussion

### 2.1. Results

In this cohort study, a subset of 744 men (30.9%) was identified as statin users for more than three years, and 1496 men (62.1%) were classified as having HSC. The HSC rate among statin users was 47.3% (352/744), while it was 68.7% (1144/1664) among the non-statin users. The overall PCa detection rate was 35.2% (848/2408), and the rate of HGPCa detection was 28.3% (240/848).

The median level of serum cholesterol levels (SC) was 222 mg/dL in men with PCa and 211 mg/dL in those without PCa, *p* < 0.001. The rate of chronic statin use, defined as more than three years of prior statins treatment, was 27.4% in men with PCa and 32.8% in men without PCa, *p* < 0.001. The age and serum prostate-specific antigen (PSA) were significantly higher in men with PCa. The testosterone level was significantly decreased in men with PCa, while the body mass index was found to be similar in both groups (Table 1).

**Table 1:** Clinical characteristics according to the diagnosis of prostate cancer.

Clinical Feature	Without PCa	With PCa	*p* Value
Age, years *	67 (46–86)	70 (52–86)	0.001
Body mass index *, kg/m^2^	27.5 (18.4–45.0)	27.7 (19.0–46.0)	0.524
Serum PSA *, ng/mL	6.9 (0.7–43.7)	7.3 (2.8–1280)	0.001
Serum cholesterol *, mg/dL	211 (101–303)	222 (101–309)	0.001
Statins use, *n*° (%)	512/1560 (32.8)	232/848(27.4)	0.006

*, values expressed in median (range).

The median level of SC was 204 mg/dL in men with PCa and 225 in those without PCa, *p* < 0.001. The rate of statin use was 33.3% in men with PCa and 25% in men without PCa, *p* < 0.017. The age, serum PSA and body mass index were significantly higher in men with PCa, while the testosterone level was similar in both groups (Table 2).

**Table 2:** Clinical characteristics according to the diagnosis of high-grade prostate cancer.

Clinical Feature	Without HGPCa	With HGPCa	*p* Value
Age, years *	68 (52–83)	74 (53–86)	0.001
Body mass index *, kg/m^2^	27.2 (20.7–46.0)	27.8 (19.0–34.8)	0.008
Serum PSA *, ng/mL	6.3 (2.8–69.6)	10.7 (3.0–1280)	0.001
Serum cholesterol *, mg/dL	225 (101–309)	204 (142–303)	0.001
Statins use, *n*° (%)	152/608 (25.0)	80/240 (33.3)	0.017

*, values expressed in median (range).

A binary logistic regression analysis was conducted, in order to determine whether SC levels and statin use were independent predictors of PCa and HGPCa. Age, serum PSA, serum testosterone and body mass index were also included as co-variables. We observed that SC was an independent predictor of PCa and HGPCa detection, while statin use was not. When SC was considered as normal *vs.* high, we found that HSC increased the risk of PC detection, OR 1.870 (95% CI 1.557–2.245), *p* = 0.001. Contrarily, HSC decreased the risk of HGPCa detection, OR 0.314 (95% CI 0.226–0.436), *p* = 0.002 (Table 3).

**Table 3:** Binary logistic regression of predictors of prostate cancer and high-grade prostate cancer.

Feature	PCa		HGPCa	
	OR (95% CI)	*p* Value	OR (95% CI)	*p* Value
Age, years	1.068 (1.050–1.087)	0.001	1.128 (1.091–1.167)	0.001
Serum PSA, ng/mL	1.021 (1.005–1.038)	0.011	1.190 (1.142–1.240)	0.001
Body mass index, kg/m^2^	1.004 (0.974–1.036)	0.785	0.983 (0.938–1.030	0.475
Serum cholesterol, (<200 *vs.* >200 mg/dL)	1.870 (1.557–2.245)	0.001	0.314 (0.226–0.436)	0.002
Statins use (no *vs.* yes)	0.876 (0.725–1.059)	0.172	1.152 (0.816–1.626)	0.421

**Figure 1:**
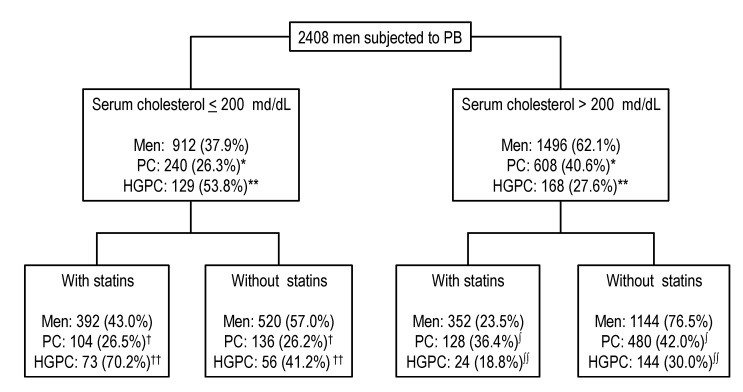
Risk of prostate cancer (PCa) detection and high-grade PCa detection according to the quality of serum cholesterol level and chronic statin use. PB, prostatic biopsy; *, *p* < 0.0001, OR: 1.917 (95% Cl 1.601–2.295); **, *p* < 0.001, OR: 0.329 (95% Cl 0.241–0.448); ^†^, *p* = 0.939, OR: 1.020 (95% Cl 0.757–1.373); ^††^, *p* < 0.001, OR: 3.263 (95% Cl 1.975–5.781); **^∫^**, *p* = 0.063, OR: 0.790 (95% Cl 0.618–1.012); **^∫∫^**, *p* < 0.011, OR: 0.538 (95% Cl 0.332–0.874).

The incidence of PCa and HGPCa is presented along with the flow rate of men, according to SC levels (first step) and then according to statins use (second step) (Figure 1). PCa was diagnosed in 240 of the 912 men with NSC (26.3%), while it was diagnosed in 608 of the 1496 men with HSC (40.6%), OR 1.917 (95% CI 1.601–2.295), *p* < 0.001. HGPCa was detected in 129 of the men with NSC (53.8%) and in 168 of the men with HSC (27.6%), OR 0.329 (95% CI 0.241–0.448), *p* < 0.001. In the subset of men with NSC, 392 (43.0%) were statin users and 520 (57.0%) were non-statin users. PCa was detected in 104 (26.5%) men and 136 (26.2%) men, respectively, OR 1.020 (95% CI 0.757–1.373), *p* = 0.939. HGPCa was detected in 73 (70.2%) men and 56 (41.2%) men, respectively, OR: 3.364, (95% CI 1.958–5.781), *p* < 0.001. In the subset of men with HSC, 352 (23.5%) were statin users and 1144 (76.5%) were non-statin users. PCa was detected in 128 (36.4%) men and 480 (42.0%) men, respectively, OR 0.790 (95% CI 0.618–1.012), *p* = 0.063. HGPCa was detected in 24 (18.8%) men and 144 (30.0%) men, respectively, OR 0.538 (95% CI 0.232–0.874), *p* = 0.011.

### 2.2. Discussion

There is already a strong foundation of basic scientific research that demonstrates how statins inhibit many pathways of cancer formation and progression through cholesterol and non-cholesterol mediated mechanisms [26]. However, those specific cholesterol-mediated mechanisms have presented a challenge in prostate cancer, especially after a study conducted on the placebo arm of the prostate cancer prevention trial. That particular study suggested that men with low cholesterol levels present a reduced risk for high-grade tumors, but not for the overall risk of prostate cancer. Nevertheless, the results in that study were not adjusted to reflect the use of cholesterol-lowering drugs [13]. A recent, nested case-control study investigating low-circulating cholesterol as a possible mechanism underlying the statins findings reported that men with low plasma cholesterol also had a lower risk for high-grade tumors, though not for an overall risk of prostate cancer. In that study, the inverse association with high-risk tumors persisted after excluding users of cholesterol-lowering drugs, suggesting that cholesterol itself may play a role.

Our study suggests the truth of the hypothesis that links the effect of statins used to reduce serum cholesterol levels to prostate carcinogenesis. For our research, we selected a population at high risk for prostate cancer detection. We mainly observed a significant association between serum cholesterol levels and the risk of prostate cancer detection. This association was observed independently of chronic statins use. This cohort had a 35% rate of prostate cancer detection, which decreased to 26% in men with low serum cholesterol levels and increased to 40% in men with high serum cholesterol levels. Moreover, the decrease in the prostate cancer detection rate was similar in men with low cholesterol levels independent of chronic statins use. These findings concur with a recent study by Murtola, *et al.*, which analyzed the influence of statins use in a Finnish cohort of 23,320 men subjected to systematic prostate cancer screening [27]. They observed a low overall prostate cancer incidence among statin users when the bias between users and non-users, due to differential PSA testing, was eliminated. In our study, we observed a 36% rate of prostate cancer detection in statin non-users with high serum cholesterol, while the rate for those men with high serum cholesterol who used statins was 42%. These results suggest that the role of statins in prostate cancer prevention could be restricted to those men, whose serum cholesterol levels were normalized after statins use. It was interesting to observe that our logistic regression analysis confirmed that serum cholesterol levels were an independent predictor of prostate cancer detection, though in the case of chronic statin use, they were not.

As we have observed, some epidemiological studies have found an inverse association between serum cholesterol levels and prostate cancer incidence [7,8,27]. A recent study, conducted on a cohort of 12,996 men with a follow-up of 37 years, suggested that men with high serum cholesterol are at greater risk of being diagnosed with high-grade prostate cancer tumors, though not at a greater overall risk of prostate cancer detection [15]. For this reason, we believe that a well-designed prospective study should be conducted to elucidate the exact role of statins use in prostate cancer chemoprevention.

Several mechanisms, by which cholesterol and prostate carcinogenesis may be linked, have been proposed. Prostate cancer cells tend to over-accumulate cholesterol in their cell membranes forming large lipid rafts, which may facilitate pro-carcinogenic cell signaling in the cancer cells. Moreover, several other pathways considered to be vital in carcinogenesis, such as sonic hedgehog and Akt pathways, are also cholesterol sensitive. Thus, having lower cholesterol levels may inhibit these pro-carcinogenic activities in prostate cells [28]. Our study suggests the hypothesis that statins can prevent prostate cancer development through a cholesterol-mediated mechanism.

A critical point of our study was to determine the aggressiveness of detected tumors with relation to the use of statins and the induced changes in serum cholesterol levels. We founded a disturbingly increased rate of high-grade tumors in statin users with normalized serum cholesterol, while the rate was very low in those men with high serum cholesterol who did not use statins. The interpretation of these results presents a challenge, since most of the recent reports have suggested an association between low cholesterol levels and a lesser probability of high-grade tumors [11,12,13]. However, none of those studies related this fact to statin consumption. Again, it is interesting to examine the Finnish group’s study on statins use in their prostate cancer screening trial [26]. In that study, statin users had a significantly decreased risk of being diagnosed with tumors having a Gleason score of 2 to 6 with respect to non-users. However, this significant decrease was not seen in tumors with Gleason scores of 7 to 10 or advanced tumors. The Finnish group’s and our results conflict with the widely held view that statins prevent aggressive tumors. We would like to emphasize, once again, that a well-designed prospective study is needed to determine whether statins are able to prevent aggressive tumors.

Admittedly, our study suffers from the many weaknesses encountered in most case and control studies. It is not a prospective cohort and controlled study. We selected a population with a high risk for prostate cancer detection; however, due to the influence of statins in the serum PSA, a selection bias for prostate biopsy could have modified the results. The influence of some lifestyle habits, comorbidities, and other concomitant treatments in prostatic carcinogenesis can also act as confounders. The criteria for defining chronic treatment, three years, may also have introduced a bias, as well as the specific statins used and their dosage.

## 3. Patients and Methods

Patients: Between January 2006 and December 2011 we conducted a prospective study on lifestyle habits, metabolic and cardiovascular comorbidities, and concomitant treatments in 3281 Mediterranean men scheduled for prostatic biopsy (PB), due to PSA levels above 4 ng/mL and/or an abnormal digital rectal examination. All participants received detailed information about the study procedure and provided written informed consent for PB and participation in the study. All procedures were in accordance with the ethical standards established in our country. Internal review board approval was not required for such a non-experimental study. All patients were given a questionnaire and underwent physical examination (size, weight, abdominal waist measurement and blood pressure assessment) and laboratory profile screening. Patients were asked about the use and duration of statins treatment and their serum cholesterol levels (SC) were determined.

To carry out this analysis, 2408 men were selected, after excluding those men who were undergoing 5-α-reductase inhibitors treatment and those men who had been using statins for less than three years. SC was classified as normal (NSC) at <200 mg/dL and high (HSC) at >200 mg/dL [11]. PCa was detected in 848 men (35.2%), and 240 (28.3%) were HGPCa (Gleason score > 7). The overall demographics and clinical characteristics of the men enrolled are summarized in Table 4.

**Table 4:** Overall demographics and clinical characteristics of enrolled men.

Clinical Feature	Measurement
Men, no (%)	2408 (100)
Age, years *	68 (46–86)
Body mass index *	27.6 (18.4–46.0)
Serum PSA *, ng/mL	6.9 (0.7–1280)
Serum cholesterol *, mg/dL	212 (101–309)
Statins treatment (%)	744 (30.9)
Prostate cancer detection	848 (35.2)
Gleason score 8–10 (%)	240 (28.3%)

*, values expressed in median (range).

Prostate biopsy technique: Transrectal ultrasound guided PB was performed as an outpatient procedure under local anesthesia. An end-fire ultrasound transducer (Falcon 2101, B-K Medical, Inc., Peabody, MA, USA) and a 16-gauge automated biopsy needle (Bard, Inc., Covington, GA, USA) were used. A minimum of 10 cores were obtained, and from 2 to 8 additional ones were taken based on age and prostate volume, according to a modified Vienna nomogram [29].

Statistical analysis: Quantitative variables were expressed as medians + semi-interquartile range (range). Qualitative variables were expressed as rates. Univariate analysis included the Chi-square test to analyze the association between qualitative variables and the Cochran test to evaluate their strength. The Mann–Whitney U test was performed to compare quantitative variables. Multivariate analysis using the binary logistic regression was carried out to examine the independent predictors of PCa risk and tumor aggressiveness. Odds ratios (OR) and 95% CI were calculated. The SPSS program V.20 (IBM, Chicago, IL, USA) was used to perform this statistical analysis.

## 4. Conclusions

We would like to conclude with the following hypothesis: Statins may prevent prostate cancer development by lowering cholesterol levels. Tumor aggressiveness may be related to the effectiveness of statins in normalizing cholesterol levels. In conjunction with our hypothesis, we would like to include this stipulation: In the future, a prospective and well-designed study considering statins consumption, as well as cholesterol levels, is required in order to clarify the specific role of statins in the chemoprevention of prostate cancer.

## Supplementary Files

Supplementary File 1
